# From public digital discourse to sustained engagement: Digital cultural translation of the Twenty-Four Solar Terms

**DOI:** 10.1371/journal.pone.0361505

**Published:** 2026-10-27

**Authors:** Jiafeng Jiang, Yuchen Song

**Affiliations:** School of Design, Silla University, Busan, Republic of Korea; Bahir Dar University, ETHIOPIA

## Abstract

Digital media can increase the visibility of living heritage without securing understanding or sustained engagement. We examined how public digital discourse about the Twenty-Four Solar Terms can be translated into an interpretive stimulus and how users evaluate it. We collected 3,433 publicly visible online text units and retained 1,101 after cleaning and anonymisation. The retained corpus informed collaborative thematic reading; 1,089 texts met the token threshold for keyword extraction and auxiliary topic-model inspection. Thematic analysis identified six cultural theme categories. They covered seasonal diet and bodily care; temporal order and heritage knowledge; and natural phenology and seasonal perception. They also covered folk customs and ritual life; aesthetic imagery and digital creative expression; and agricultural production and everyday practice. These categories informed a five-page storyboard through theme-design mapping and expert-assisted review. Survey data from 350 valid respondents were analysed with covariance-based structural equation modelling (CB-SEM) and supplementary fuzzy-set qualitative comparative analysis (fsQCA). Information clarity, visual aesthetics, perceived explorability and perceived authenticity were positively associated with perceived cultural value. Perceived explorability had the largest standardised path estimate for cultural value, and perceived authenticity was also associated with cultural identity. Perceived cultural value and cultural identity were associated with sustained cultural engagement intention. Configurational analysis identified four sufficient configurations for high engagement and four for low engagement. The two solution sets were asymmetric rather than mirror images. These findings link perceived interpretive qualities, cultural value and cultural identity with sustained cultural engagement intention.

## Introduction

Digital media are changing how people encounter cultural heritage. Short videos, websites, digital exhibitions, online museums and cultural-creative interfaces now bring heritage content to everyday audiences. Museums, heritage sites and festival venues remain important points of contact. Research shows that digital display and virtual cultural experiences can expand access and reshape how users understand and participate in heritage [1–3]. Visibility alone, however, does not secure cultural understanding. A Solar Terms poster may be reposted, a short video watched and a digital exhibition page browsed. Yet the time knowledge, life experience and cultural value behind these materials may remain weakly understood. For living heritage, circulation matters when new media environments reactivate knowledge, practice, memory and value [4,5].

Research on participatory and interactive digital heritage [6–8] supports a distinction between digital visibility and cultural vitality. Digital visibility is the opportunity for heritage to enter public digital space and be encountered by users. Cultural vitality concerns whether users understand the heritage, find it meaningful, and intend to continue learning, experiencing, sharing, or participating [9–11]. Prior research indicates that continued engagement with digital heritage depends on experiential value, cultural identity and subsequent behavioural intention. It does not depend on technological presentation alone [3,12–14].

The Twenty-Four Solar Terms offer a representative case. In 2016, UNESCO inscribed them on the Representative List of the Intangible Cultural Heritage of Humanity [15,16]. They form a time-knowledge system organised around the sun’s annual motion, seasonal change, agricultural experience, dietary customs, bodily perception and family memory. In contemporary digital media, Solar Terms culture appears in posters, short videos, online exhibitions, cultural-creative interfaces and public cultural communication. Content that presents only Solar Terms names, seasonal colours or traditional illustrations may let users see the Solar Terms. Without further interpretation, however, users may not understand the relationships among natural change, bodily rhythm, dietary life and family memory. The challenge is to organise these relationships into digital materials that are understandable, explorable and credible.

Existing studies have examined how traditional culture enters modern design through cultural design, symbolic transformation and sustainable communication [17,18]. Research on digital museums and virtual cultural experiences likewise shows that digital media can influence user experience, cultural identity and subsequent participation [8,9,19]. Work on mixed reality, digital cultural tourism and immersive experience suggests that media form alone does not generate cultural value. Continued participation often depends on clear explanatory cues, accessible experiential paths and credible cultural contexts [10,11]. For the Twenty-Four Solar Terms, few studies have examined the full chain from public digital meanings to their transformation into interpretive materials. Fewer still have linked this process to users’ value judgements, cultural identity and engagement intention.

This article uses ‘digital cultural translation’ to describe the methodological process through which cultural meanings identified in public digital discourse were transformed into a controlled interpretive stimulus. Drawing on translation, localisation and transcreation research [20–22], the process comprised corpus construction, thematic analysis, theme-design mapping, expert review and development of the five-page storyboard. The participant-level Stimulus–Organism–Response (S-O-R) model examined users’ evaluations of the stimulus. It focused on information clarity, visual aesthetics, perceived explorability and perceived authenticity. The model also assessed their associations with perceived cultural value, cultural identity and sustained cultural engagement intention.

This article treats the Twenty-Four Solar Terms as living calendrical heritage. It examines how digital cultural translation may support sustained cultural engagement. Six research questions guide the study:

RQ1: What recurring cultural meanings of the Twenty-Four Solar Terms are represented in public digital discourse?

RQ2: How can these recurring cultural meanings be translated into a controlled digital interpretive stimulus?

RQ3: Which digital interpretive conditions are associated with perceived cultural value and cultural identity as Organism-level responses within the S-O-R framework?

RQ4: How do perceived cultural value and cultural identity link digital interpretive conditions with sustained cultural engagement intention through mediating and serial pathways?

RQ5: Which configurations of conditions are empirically sufficient for high sustained cultural engagement intention?

RQ6: Which configurations of conditions are empirically sufficient for low sustained cultural engagement intention?

The study makes three contributions.

First, it situates the Twenty-Four Solar Terms within living calendrical heritage. Reading the Solar Terms through time knowledge, natural observation, bodily experience and everyday memory moves digitisation research beyond formal presentation towards the interpretation of cultural meaning.

Second, it uses digital cultural translation to organise stimulus development. Public digital discourse, thematic analysis, design mapping, expert review and user evaluation are linked in one process. This linkage makes the movement from cultural materials to digital design more transparent. It also offers an analytical path for research on traditional-culture digitisation.

Third, it combines a variable-oriented S-O-R model with configurational analysis to examine net relationships and alternative combinations associated with sustained cultural engagement. The two approaches distinguish average relational pathways from configurational equifinality and asymmetry.

## Literature review and research hypotheses

### Living calendrical heritage and digital cultural translation

The Twenty-Four Solar Terms form a time-knowledge system organised around solar motion, seasonal rhythm, agricultural experience, dietary practice, bodily perception and family memory [16,17]. Their heritage significance lies in more than twenty-four calendrical names. It also lies in the continued interpretation and enactment of seasonal knowledge through everyday practices. This combination of structured knowledge, embodied experience and cultural memory supports their treatment as living heritage rather than as a fixed symbolic catalogue [4,5].

From a digital heritage perspective, the issue is how living heritage is interpreted after entering digital media [9–11]. Public platforms, short videos, websites and digital exhibitions can make Solar Terms culture more visible [8]. Without clear information structure, lived experience and credible cultural context, however, digital presentation may remain at the level of seasonal names, illustrations or symbolic decoration [17,18]. Digital heritage research shows that authenticity, participation and cultural identity do not arise automatically online. They require concrete interpretive structures [23]. A cultural translation experience that organises seasonal knowledge, phenological observation, dietary habits, bodily perception, family memory and contemporary participation into understandable, explorable paths may therefore support sustained engagement.

### S-O-R mechanisms in digital heritage evaluation

S-O-R theory argues that external environmental stimuli influence behavioural responses through individuals’ internal states [14,24–26]. In digital heritage contexts, stimuli include interface visuals and technological features, as well as cultural organisation, information clarity, authenticity cues and explorable structures. Studies on digital cultural heritage and virtual museums report associations among experience quality, perceived value, cultural identity and continuous behavioural intention [3,12–14].

At the participant level, the S-O-R model comprises information clarity, visual aesthetics, perceived explorability and perceived authenticity as Stimulus evaluations. Perceived cultural value and cultural identity constitute the Organism. Sustained cultural engagement intention constitutes the Response.

### Complementary use of CB-SEM and fsQCA

Covariance-based structural equation modelling (CB-SEM) and fuzzy-set qualitative comparative analysis (fsQCA) address different parts of the research problem. CB-SEM estimates associations among latent variables and tests the proposed mediation relationships. FsQCA examines whether different combinations of conditions are sufficient for the same outcome. It also tests whether configurations for high engagement differ from those for low engagement [27–30].

### Digital interpretive conditions and perceived cultural value

Information clarity concerns whether the knowledge structure and cultural relationships of the Twenty-Four Solar Terms are presented understandably. Heritage interpretation research suggests that organised and accessible information can support attention, comprehension and active meaning-making [31,32]. Visual aesthetics concerns users’ perceptions of presentation coherence and attractiveness. Previous research indicates that perceived visual aesthetics contributes to the accessibility and experiential quality of digital interfaces and cultural content [10,19,33].

Perceived explorability concerns whether the stimulus suggests further layers, connections or paths of cultural discovery. Experience-design and heritage-interpretation research indicates that meaningful engagement strengthens when users move beyond passive observation and see opportunities for continued exploration [34–36]. Perceived authenticity concerns whether users believe that the digital presentation maintains a credible relationship with the cultural context, knowledge sources and lived meanings of the Twenty-Four Solar Terms [23,37]. Understandable, aesthetically coherent, explorable and culturally credible materials may help users recognise contemporary cultural value [9,12,19]. The following hypotheses are therefore proposed:

H1a. Information clarity is positively associated with perceived cultural value.

H1b. Visual aesthetics is positively associated with perceived cultural value.

H1c. Perceived explorability is positively associated with perceived cultural value.

H1d. Perceived authenticity is positively associated with perceived cultural value.

### Authenticity, cultural value, and cultural identity

Cultural identity concerns whether users understand the Twenty-Four Solar Terms as a cultural resource related to their own cultural experience, life memory and sense of self [38,39]. In digital heritage experiences, authenticity is more than historical appearance. It also concerns whether cultural expression is credible, retains context and remains related to living practice [37,40–42]. When users believe that a digital presentation follows the basic logic of Solar Terms culture, they may incorporate its cultural meaning into their own understanding and form cultural identity.

Perceived cultural value is users’ judgement of whether digital content about the Twenty-Four Solar Terms has symbolic, educational, aesthetic, practical and social significance today. When users perceive heritage as meaningful and relevant to contemporary life, they may connect it with their own cultural experience and sense of belonging [9,38,39]. Perceived cultural value may therefore strengthen cultural identity. The following hypotheses are proposed:

H2. Perceived authenticity is positively associated with cultural identity.

H3. Perceived cultural value is positively associated with cultural identity.

### Sustained cultural engagement and mediation mechanisms

Sustained cultural engagement intention refers to users’ willingness to continue browsing, learning about, experiencing, sharing, or participating in related cultural content after encountering digital materials about the Twenty-Four Solar Terms. Previous digital museum and virtual heritage studies have examined related post-experience outcomes through continuance, recommendation, revisit, or participation intentions [9,12,13].

Within the S-O-R framework, perceived cultural value and cultural identity represent two internal evaluations through which the interpretive stimulus may be associated with engagement intention [14,24–26]. Users who perceive the Twenty-Four Solar Terms as culturally valuable and identity-relevant may therefore be more willing to maintain contact with related knowledge, practices, and digital content [9,38,39]. The following hypotheses are proposed:

H4. Perceived cultural value is positively associated with sustained cultural engagement intention.

H5. Cultural identity is positively associated with sustained cultural engagement intention.

Digital interpretive conditions may be associated with engagement indirectly through cultural value and identity formation. The interpretive stimulus first provides conditions under which cultural meaning can be understood and evaluated. Perceived cultural value may then support identity connection, which may further strengthen engagement intention [9,13]. Therefore, this article proposes the following mediation hypotheses:

H6a. Perceived cultural value mediates the relationship between digital interpretive conditions and sustained cultural engagement intention.

H6b. Cultural identity mediates the relationship between perceived cultural value and sustained cultural engagement intention.

H6c. Perceived cultural value and cultural identity serially mediate the relationship between digital interpretive conditions and sustained cultural engagement intention.

## Materials and methods

### Ethics approval, participants, and survey procedure

The Ethics Committee of Nanjing Normal University approved this study (Approval No. NNU202603082; approval date: March 27, 2026). We administered the formal online questionnaire through the Wenjuanxing online survey platform (www.wjx.cn) on April 11–12, 2026. Participants had to be at least 18 years old, reside in mainland China, read the Chinese questionnaire and confirm that they had fully viewed the five-page stimulus. Before accessing the survey items, they read the study information and informed consent statement. They then provided electronic informed consent. Participation was voluntary, participants could withdraw at any time, and no personally identifiable information was collected.

Participants first confirmed informed consent. They then viewed the storyboard-based digital stimulus and completed items on digital interpretive conditions, perceived cultural value, cultural identity and sustained cultural engagement intention. Finally, they provided demographic and background information.

All 383 questionnaire responses entered the final data-quality screening stage. We evaluated responses with the embedded attention-check item and additional criteria for mechanical or patterned responding and implausibly short completion times. Thirty-three responses failed one or more criteria and were excluded. The final analytical sample comprised 350 valid cases for both the CB-SEM and fsQCA analyses.

### Overall research design

This study organised a five-stage sequential multi-method workflow linking the contemporary cultural expression of the Twenty-Four Solar Terms with digital interpretation and sustained cultural engagement. The sequence was informed by Creswell and Plano Clark’s exploratory sequential design [43], in which findings from an initial interpretive component provide a foundation for subsequent quantitative inquiry. In this study, cultural findings informed the development of a digital research stimulus, while the questionnaire measures were adapted from established scales. Bazeley’s principles of mixed-method integration [44], building integration [45] and the pathway-building perspective [46] informed the connections among cultural evidence, stimulus development and participant evaluation. Creswell and Inoue’s approach to mixed-method data analysis further supports the alignment of evidence and interpretation across these research components [47]. The five stages operationalised this integrated research sequence.

Stage 1 established the contemporary cultural evidence base through construction and preprocessing of a public digital corpus. The Twenty-Four Solar Terms are living cultural heritage expressed through seasonal knowledge, everyday practices, collective memory, cultural narratives and contemporary communication [4–8,15–18]. Research on digital heritage communication and social-media perceptions of intangible heritage shows how cultural meanings are expressed and renewed in public digital environments [48,49]. We therefore collected publicly accessible Chinese-language materials to ground the stimulus in contemporary cultural discourse. After screening, cleaning and duplicate removal, 1,101 records were retained as the empirical basis for thematic interpretation.

Stage 2 identified recurring cultural meanings through interpretive thematic analysis supported by computational text analysis. Braun and Clarke’s thematic-analysis framework guided the identification of patterned meanings in context [50], while Krippendorff’s content-analysis principles supported systematic comparison of textual evidence [51]. The research team jointly read the retained texts, grouped recurring expressions by cultural meaning and refined the emerging categories through discussion. Term frequency–inverse document frequency (TF-IDF) weighting identified comparatively distinctive expressions [52], and latent Dirichlet allocation (LDA) was used to inspect topic–word patterns within the corpus [53]. These complementary forms of evidence informed the interpretive synthesis of six cultural themes.

LDA provided an interpretable probabilistic representation of lexical associations that could be examined alongside TF-IDF outputs and contextual reading. Methodological developments in topic modelling, including embedding-based approaches and comparative assessments of different models, informed the choice of this supporting analytical technique [54,55]. The six cultural themes were developed through the research team’s synthesis of the textual evidence and supplied the substantive cultural content for the subsequent design stage.

Stage 3 translated the six cultural themes into a standardised five-page digital storyboard through theme-to-design mapping and expert-assisted review. Cross’s concept of designerly ways of knowing provides a basis for transforming interpreted knowledge into communicable design representations [56]. A theme-to-design matrix linked the identified meanings with informational, visual, experiential and participatory components. Nine reviewers with relevant design and digital-heritage expertise assessed these correspondences, and their feedback informed refinement of the storyboard. This stage applied building integration by connecting cultural interpretation directly with the stimulus used in participant evaluation [45,46]. Stages 1–3 constituted the digital cultural translation process.

Stage 4 evaluated the completed storyboard through the Stimulus–Organism–Response (S-O-R) framework [24–26]. The model links perceptions of a stimulus with internal appraisals and subsequent behavioural intentions, matching the study’s interest in cultural interpretation and continuing engagement. Information clarity, visual aesthetics, perceived explorability and perceived authenticity represented evaluations of the Stimulus; perceived cultural value and cultural identity represented the Organism; and sustained cultural engagement intention represented the Response. Research on technology-related intentions [57,58] and digital-heritage experiences [3,12–14,42] provided further context for examining participant responses to the digital presentation. The same storyboard was evaluated through the formal questionnaire, and quality screening of 383 submissions yielded a final analytical sample of N = 350.

Stage 5 examined the participant-level evidence through covariance-based structural equation modelling (CB-SEM) and fuzzy-set qualitative comparative analysis (fsQCA). CB-SEM tested the hypothesised latent-variable relationships and mediation pathways using Anderson and Gerbing’s two-step approach to measurement and structural model evaluation [59]. Because the aim was to assess a theoretically specified model, the analysis included global fit measures such as CFI, TLI, RMSEA and SRMR, following established SEM guidance [60,61] and methodological work on the purposes of different SEM approaches [62].

fsQCA provided a complementary analysis of how combinations of conditions relate to sustained cultural engagement. Drawing on Ragin’s set-theoretic approach [63] and the principles of conjunctural causation, equifinality and causal asymmetry [27–29], the analysis identified alternative sufficient configurations associated with high and low engagement intention. CB-SEM thus examined the proposed relational and mediation structure, while fsQCA examined the multiple combinations of conditions associated with the outcome.

Together, the five stages formed a continuous research process in which public digital cultural materials were interpreted into recurring themes, translated into a common digital stimulus and evaluated by participants. The resulting survey evidence was then examined through complementary relational and configurational analyses. The complete research process is presented in Fig 1.

**Fig 1:**
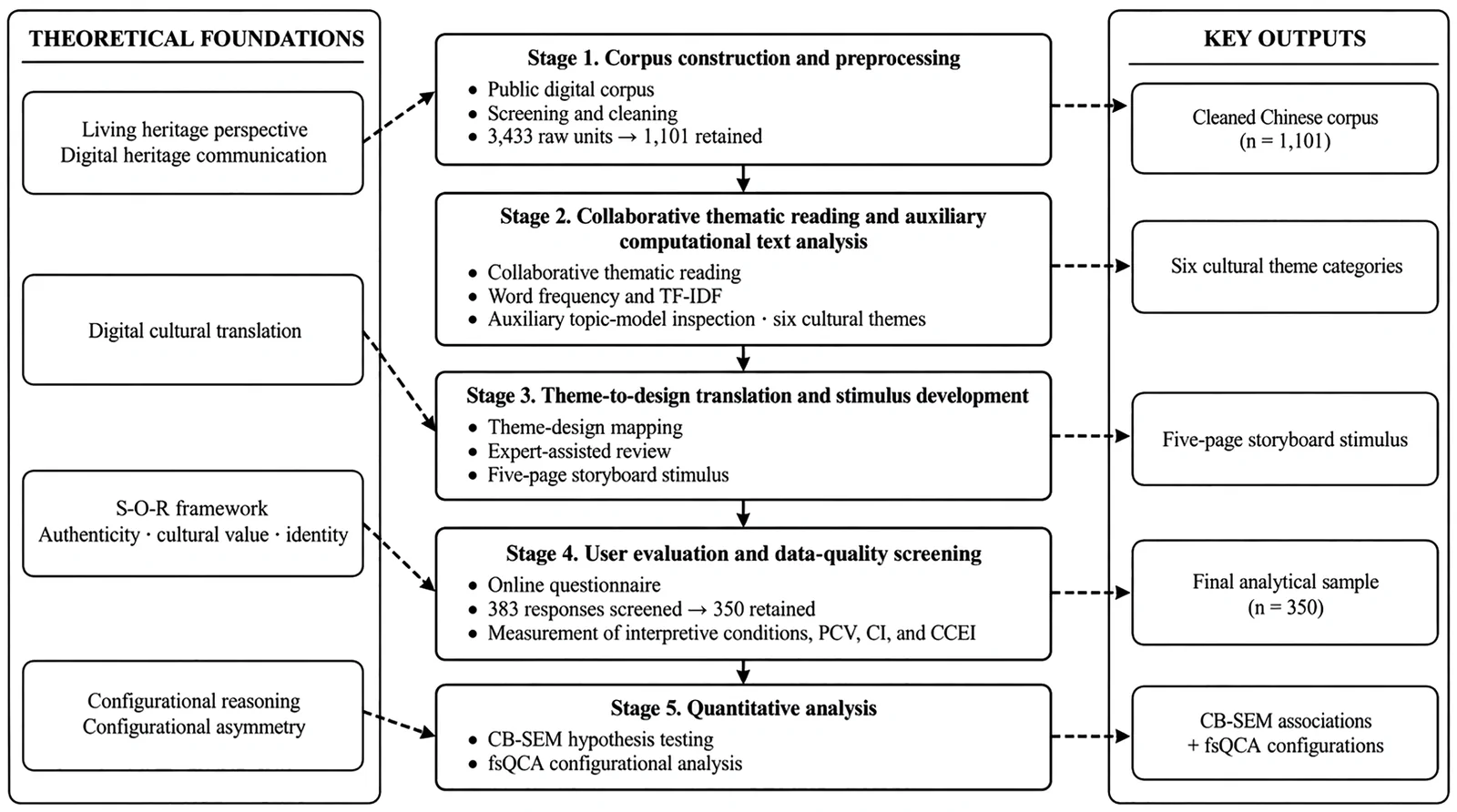
Five-stage sequential multi-method workflow for digital cultural translation and participant-level evaluation. Stages 1-3 transform the public corpus into the stimulus; Stage 4 screens 383 survey responses to a final N = 350; Stage 5 combines CB-SEM and fsQCA.

### Public digital text corpus and data collection

The public digital corpus consisted of publicly visible Chinese-language materials on the Twenty-Four Solar Terms. We retrieved the materials in March 2026 with 21 keyword combinations covering the Solar Terms system and the Four Beginnings (Lichun, Lixia, Liqiu and Lidong). The retrieval strings are listed in S1 Appendix. Item-level retrieval timestamps were not retained. The field publish_date_or_access_date records the dataset curation date (June 1, 2026), not the publication date or retrieval date of each source.

Sources included official cultural and government websites, such as People.cn, Xinhuanet, CCTV and the Ministry of Culture and Tourism. They also included news portals, encyclopedia, health-science, academic, design, social and commercial pages (Table 1). We excluded login-protected, paywalled and private content. Personal identifiers were not retained.

**Table 1:** Composition of the public online corpus.

Source category	Original units	Retained units	Retention rate
Health popularisation pages	1,535	525	34.2%
Article pages	624	142	22.8%
Official cultural websites	518	193	37.3%
Public design/cultural pages	488	136	27.9%
Academic abstract pages	244	84	34.4%
Forum/social search snippets	11	11	100.0%
E-commerce search snippets	10	7	70.0%
Encyclopedia pages	3	3	100.0%
Total	3,433	1,101	32.1%

Note: Source categories were assigned at retrieval.

Of the 3,433 raw text units, 2,202 were excluded as unrelated to the Solar Terms. A further 80 were excluded as advertising or site noise, and 50 as exact duplicates. This process left 1,101 anonymised units for analysis (screening rules in S1 Appendix and S2 Code).

### Thematic analysis of public digital discourse on the Twenty-Four Solar Terms

We used an interpretive thematic reading of the public texts to identify recurring cultural meanings for stimulus development, drawing on principles of patterned-meaning analysis [50] and transparent textual interpretation [51]. The research team jointly read the retained Chinese-language records in context and discussed recurring expressions concerning seasonal knowledge, practices and cultural meanings. Related expressions were grouped by shared meaning, and these groupings were reviewed and refined through further reading and discussion to form six cultural theme categories. This procedure was a collaborative thematic synthesis for design development; it did not involve independent coder classifications or intercoder agreement testing. Table 3 illustrates the relationship between representative expressions and the resulting categories, and S1 Appendix explains their use in the storyboard.

We first segmented the cleaned Chinese texts with jieba and then removed tokens shorter than two characters, tokens on the stop-word list and tokens containing no Chinese characters. Documents with fewer than three remaining tokens were excluded from computational text analysis. Re-execution of the supplied script on the retained 1,101-record Chinese corpus excluded 12 such documents, leaving 1,089 for word-frequency analysis, term frequency–inverse document frequency (TF-IDF) keyword extraction [52] and auxiliary latent Dirichlet allocation (LDA) topic inspection [53]. The 1,101-record corpus remained the basis for the team’s thematic reading. Lexical and topic outputs informed that reading but did not determine the final categories. Analyses used Python with pandas, jieba and scikit-learn; parameters and the verified re-execution environment are documented in S1 Appendix and S2 Code. In S2 Code, TF-IDF used scikit-learn’s TfidfVectorizer (min_df = 1). Auxiliary LDA used scikit-learn’s LatentDirichletAllocation fitted to a raw term-count matrix generated with CountVectorizer (min_df = 1), with batch learning and random_state = 42. The code did not explicitly set max_df or max_features for either vectorizer, so their library defaults applied. Solutions with K = 4–8 were inspected using the 12 highest-weighted words per topic; the code did not select an optimal K using coherence or perplexity. The Chinese stop-word list is included in S2 Code. The exact package versions used in the original analysis were not recorded; the versions documented in S2 Code refer to the verified re-execution environment.

### Expert review and theme-design translation

After the thematic analysis, the research team developed a theme-design translation matrix linking the identified cultural themes to storyboard components. Nine reviewers with backgrounds in cultural creative design, visual and interaction design, digital heritage communication and design research methods reviewed the mappings. The research team revised mappings that received repeated concerns and discussed them again [56]. No personal information was collected from the reviewers. Their comments were used only to revise the stimulus. The final mapping informed the storyboard used in the questionnaire.

### Questionnaire design and variable measurement

The questionnaire consisted of two parts. The first measured the seven model constructs with reflective items adapted from prior studies and revised for the Solar Terms context. Items used a seven-point Likert scale (Table 2; full wording in S1 Appendix).

**Table 2:** Measurement structure, scale items, and reference sources.

Code	Measurement structure	Item focus (full wording in S1 Appendix)	Author(s) and year	Theoretical/ source basis	Reference no.
IC	Stimulus/ interpretive condition	Four reflective items on recognising the core theme, information clarity, knowledge understandability, and information organisation.	Trunfio et al., 2022; Chen et al., 2024; Tilden, 1957; Moscardo, 1996	Digital heritage experience and information clarity; heritage interpretation and mindful visitor research	[10,19,31,32]
VA	Stimulus/ interpretive condition	Four reflective items on visual comfort, colour-layout coordination, thematic consistency, and overall aesthetic impression.	Trunfio et al., 2022; Chen et al., 2024; Lavie and Tractinsky, 2004; Norman, 2013	Digital heritage experience; perceived visual aesthetics of websites; design perception and everyday design experience	[10,19,33,64]
PE	Stimulus/ interpretive condition	Four reflective items on further knowledge cues, exploratory prompts, perceived depth, and connections among seasons, everyday life, and culture.	Pine and Gilmore, 1998; Schmitt, 1999; Hassenzahl, 2010	Experience economy; experiential marketing; experience design and exploratory engagement	[34–36]
PA	Stimulus/ cultural evaluation condition	Four reflective items on cultural preservation, authentic cultural meaning, everyday-life context, and the cultural spirit of the Solar Terms.	Wang, 1999; Kolar and Zabkar, 2010; Cohen, 1988; Jones et al., 2018	Tourism authenticity; consumer-based authenticity; authenticity and commoditisation; digital heritage authenticity	[23,37,40,41]
PCV	Organism/ cultural value evaluation	Four reflective items on contemporary cultural meaning, life value, modern relevance, and the need for sustained transmission.	Deng et al., 2023; Jiang et al., 2024; Meng and Dolah, 2025	Perceived value and cultural identity in digital museuming; continuous behavioural intention in virtual heritage tourism; virtual museum experience quality and sustainable heritage engagement	[9,12,13]
CI	Organism/ cultural identity	Four reflective items on perceived closeness, identification with seasonal culture, personal relevance, and cultural connection.	Deng et al., 2023; Hall, 1996; Tajfel and Turner, 1979	Cultural identity in digital museuming; cultural identity theory; social identity theory	[9,38,39]
CCEI	Response/ sustained cultural engagement intention	Four reflective items on continued learning, following related digital or cultural activities, sharing/recommending content, and using Solar Terms knowledge in daily life.	Trunfio et al., 2022; Deng et al., 2023; Chen et al., 2024; Jiang et al., 2024; Meng and Dolah, 2025	Post-experience behaviour in museum mixed reality; digital museuming and visit intention; AR heritage museum use; continuous behavioural intention in virtual heritage tourism; virtual museum engagement and offline visit intention	[9,10,12,13,19]

Note. IC = information clarity; VA = visual aesthetics; PE = perceived explorability; PA = perceived authenticity; PCV = perceived cultural value; CI = cultural identity; CCEI = sustained cultural engagement intention.

The second part collected demographic and background variables, including gender, age, education, occupation, prior knowledge of the Twenty-Four Solar Terms, and prior exposure to digital cultural exhibitions. The questionnaire also contained an embedded attention-check item.

### Common method bias and data screening

Because the survey used self-reported data collected in a single measurement session, we took several steps to reduce common method bias. The questionnaire stated that participation was voluntary and anonymous. It also stated that there were no right or wrong answers and that responses would be used only for academic research. Stimulus viewing and scale responses were arranged in separate sections. Attention checks were used to identify low-quality responses. At the statistical level, we compared the theoretical seven-factor confirmatory factor analysis (CFA) model with a one-factor CFA model. We also examined discriminant validity, model fit and collinearity diagnostics. The latter included full-collinearity variance inflation factors (VIFs) computed from construct mean scores [65,66].

### Analytical strategy

First, CB-SEM was the primary method for testing the hypothesised measurement and structural relationships. We estimated the models using the CB-SEM module of SmartPLS 4.1.0.9 with maximum-likelihood estimation. We scaled each latent variable by fixing its first loading to 1. Seven-point Likert indicators were treated as approximately continuous. The dataset contained no missing values. We examined distributional assumptions with item-level skewness, excess kurtosis and Mardia’s tests of multivariate skewness and kurtosis [60]. We evaluated the measurement model with standardised factor loadings, Cronbach’s alpha, composite reliability (CR), average variance extracted (AVE) [59,67] and the heterotrait-monotrait ratio (HTMT) [68,69]. The conventional guidelines were alpha and CR >=.70, AVE >=.50 and HTMT < .85. We evaluated overall fit with chi-square/df, comparative fit index (CFI), Tucker-Lewis index (TLI), root mean square error of approximation (RMSEA) and standardised root mean square residual (SRMR). The fit guidelines were chi-square/df < 3, CFI/TLI >=.90 and RMSEA/SRMR <=.08 [60,61]. Because maximum-likelihood standard errors can be affected by multivariate non-normality, we evaluated direct and indirect associations with 5,000 nonparametric bootstrap resamples. We report standardised estimates with bootstrap standard errors and 95% bias-corrected bootstrap confidence intervals (BC CIs) [70]. The complete model specification is provided in S1 Appendix.

Second, we conducted fsQCA in fsQCA 4.1 as a complementary configurational analysis based on construct mean scores. The six conditions were information clarity, visual aesthetics, perceived explorability, perceived authenticity, perceived cultural value and cultural identity. Sustained cultural engagement intention was the outcome. Direct calibration used the 95th, 50th and 5th sample percentiles as the anchors for full membership, the crossover point and full non-membership, respectively. Anchor values are provided in S1 Appendix.

We set memberships of exactly 0.500 to 0.501 so that cases at the crossover point were not dropped from truth-table rows [27]. Results with unadjusted 0.500 memberships are reported as a sensitivity analysis. A condition was considered necessary only when necessity consistency reached.90. For sufficiency analysis, the frequency threshold was 3 cases. A truth-table row was coded as sufficient only when raw consistency was at least.80 and proportional reduction in inconsistency (PRI) consistency was at least.65. Rows that met the raw-consistency threshold but fell below the PRI threshold were treated as contradictory and coded as not sufficient. Under this rule, no truth-table row was sufficient for both high and low engagement.

We analysed high and low engagement separately to evaluate configurational asymmetry. For the intermediate solution, each condition was expected to contribute to high engagement when present and to low engagement when absent. A condition was classified as core when it appeared in both the parsimonious and intermediate solutions. It was classified as peripheral when it appeared only in the intermediate solution [27–29,63]. We used a stricter PRI threshold of.70 as a robustness check. The truth tables of all observed configurations, complex, intermediate and parsimonious solutions, and sensitivity results are provided in S1 Appendix.

## Results

### Cultural themes in public digital discourse

Thematic analysis identified six recurring cultural themes related to the Twenty-Four Solar Terms (Table 3).

**Table 3:** Semantic grouping, theme integration, and implications for digital heritage design.

Analytic step	Representative semantic units	Integrated cultural theme category	Interpretive implication for digital heritage design
Semantic grouping	“eating spring pancakes”, “tasting the fresh”, “Lixia eggs”, “autumn nourishment”, “winter nourishment”, “health preservation in accordance with the season”	Seasonal diet and bodily care	Connects seasonal time, bodily adjustment, family diet, and health-preservation practices.
Semantic grouping	“twenty-four nodes”, “annual cycle”, “spring-summer-autumn-winter transitions”, “agricultural calendar”, “observing phenomena and regulating time”	Temporal order and heritage knowledge	Presents the Solar Terms as a readable time-knowledge system rather than a list of names.
Semantic grouping	“the east wind thaws”, “insects stir at Jingzhe”, “white dew”, “frost descent”, “snowfall”, “plants sprouting”	Natural phenology and seasonal perception	Uses natural signs as sensory evidence for perceiving seasonal transition.
Semantic grouping	“welcoming spring”, “biting spring”, “sun-drying autumn harvests”, “winter sacrifice”, “elder reminders”, “seasonal habits”	Folk customs and ritual life	Shows how seasonal change enters ritual, family memory, and shared everyday life.
Semantic grouping	“spring green”, “summer lotus”, “golden autumn”, “snow scenery”, “poetic atmosphere”, “poster style”	Aesthetic imagery and digital creative expression	Supports contemporary visual organisation while avoiding imagery detached from seasonal meaning.
Semantic grouping	“sowing season”, “harvest season”, “field labour”, “grain growth”, “rural courtyard”	Agricultural production and everyday practice	Maintains the relationship among calendrical knowledge, agricultural labour, and everyday life.

### From themes to design elements

Each theme was mapped to design elements (S1 Appendix, Table A1). Expert review then led to targeted revisions in knowledge structure, bodily practice cues, phenological presentation, ritual memory, visual imagery and participatory elements (Table 4).

**Table 4:** Expert review concerns and final stimulus revisions.

Review concern	Cultural theme category	Final stimulus revision
Knowledge structure	Temporal order and heritage knowledge	Page 1 introduces the Twenty-Four Solar Terms as a calendrical-cultural system and adds annual-cycle navigation.
Bodily and everyday practice	Seasonal diet and bodily care	Page 3 connects the Four Beginnings with dietary customs and bodily rhythms.
Phenological cues	Natural phenology and seasonal perception	Page 2 presents seasonal transitions through phenological symbols, seasonal cue cards, and agricultural memory.
Ritual memory	Folk customs and ritual life	Page 4 includes folk practices, family memory, and everyday practice examples.
Visual expression and participation	Aesthetic imagery and digital creative expression	Page 5 strengthens poetic imagery, contemporary interface expression, and prompts for continued exploration.
Agricultural everyday practice	Agricultural production and everyday practice	Pages 2 and 4 retain agricultural memory, seasonal labour, and everyday practice content as support.

### Stimulus design

Based on thematic analysis and expert mapping review, the final stimulus was organised as a five-page storyboard. The design used the Four Beginnings as representative seasonal transition nodes to retain the annual cycle within a short stimulus. Page 1 introduced the Twenty-Four Solar Terms as a calendrical and cultural system. Page 2 presented phenological symbols and agricultural memory. Page 3 connected the Four Beginnings with dietary customs and bodily rhythms. Page 4 presented folk practices, family memory and everyday life. Page 5 translated poetic imagery and participatory communication into contemporary interface logic and invited users to continue exploring.

The stimulus was presented as a fixed, non-interactive sequence so that all participants saw the same content in the same order; the full storyboard rationale is provided in S1 Appendix. The research team assembled the five-page storyboard using Microsoft PowerPoint and Adobe Photoshop with selected visual materials from publicly accessible online sources; its content and arrangement followed the theme-to-design matrix and expert review.

### Sample profile

The final valid sample comprised 350 cases and included variation in gender, age, education, occupation, prior Solar Terms knowledge, and digital exhibition experience (Table 5).

**Table 5:** Sample profile.

Characteristic	Distribution
Gender	Male: 172 (49.1%); female/other: 178 (50.9%)
Age	18-30 years: 231 (66.0%); 31–40 years: 58 (16.6%); 41 years and above: 61 (17.4%)
Education	Junior college or below: 163 (46.6%); bachelor’s degree or above: 187 (53.4%)
Occupation	Enterprise/public institution employees: 210 (60.0%); students: 70 (20.0%); other occupations: 70 (20.0%)
Region	Covered seven major regions of mainland China; the largest groups were North China 100 (28.6%), Southwest China 72 (20.6%), and East China 63 (18.0%)
Prior knowledge of the Solar Terms	High or very high: 213 (60.9%); moderate: 83 (23.7%); low or very low: 54 (15.4%)
Digital exhibition experience	Relatively much or very much: 206 (58.9%); moderate: 79 (22.6%); little or none: 65 (18.6%)

### Measurement model, discriminant validity, and common method bias

The measurement model showed acceptable standardised loadings (0.796–0.883), internal consistency, composite reliability, and convergent validity across all constructs (Table 6).

**Table 6:** Construct means, loadings, reliability, and AVE.

Construct	Mean	SD	Standardised loadings	alpha	CR	AVE
IC	3.897	1.180	IC1 = 0.860; IC2 = 0.870; IC3 = 0.878; IC4 = 0.841	0.920	0.921	0.743
VA	4.121	1.151	VA1 = 0.838; VA2 = 0.883; VA3 = 0.846; VA4 = 0.842	0.914	0.914	0.727
PE	3.956	1.161	PE1 = 0.856; PE2 = 0.854; PE3 = 0.826; PE4 = 0.812	0.903	0.903	0.701
PA	4.134	1.118	PA1 = 0.796; PA2 = 0.830; PA3 = 0.831; PA4 = 0.814	0.890	0.890	0.669
PCV	4.139	1.121	PCV1 = 0.823; PCV2 = 0.837; PCV3 = 0.862; PCV4 = 0.848	0.907	0.907	0.710
CI	4.023	1.172	CI1 = 0.864; CI2 = 0.859; CI3 = 0.839; CI4 = 0.858	0.916	0.916	0.731
CCEI	4.234	1.186	CCEI1 = 0.864; CCEI2 = 0.865; CCEI3 = 0.851; CCEI4 = 0.861	0.919	0.919	0.740

Discriminant validity was also supported: all HTMT values were below 0.85, with the highest value observed between PCV and CI (Table 7).

**Table 7:** HTMT matrix.

Construct	IC	VA	PE	PA	PCV	CI	CCEI
IC	–						
VA	0.710	–					
PE	0.737	0.730	–				
PA	0.719	0.688	0.754	–			
PCV	0.718	0.704	0.770	0.724	–		
CI	0.677	0.701	0.715	0.731	0.771	–	
CCEI	0.702	0.678	0.728	0.738	0.722	0.732	–

Model-fit comparisons indicated that the seven-factor CFA model fitted the data substantially better than the one-factor model, and the structural model also showed acceptable fit (Table 8).

**Table 8:** CFA, one-factor model, and structural model fit.

Model	chi-square	df	chi-square/df	CFI	TLI	RMSEA	SRMR
Seven-factor CFA model	354.176	329	1.077	0.997	0.996	0.015	0.024
One-factor CFA model	2184.762	350	6.242	0.776	0.758	0.122	0.065
Structural model	411.600	336	1.225	0.991	0.990	0.025	0.042

Item-level skewness ranged from −0.31 to 0.25 and excess kurtosis from −0.56 to −0.01, within conventional limits for approximate univariate normality [60]. Mardia’s tests nevertheless indicated multivariate non-normality (multivariate skewness χ^2^ = 4702.48, df = 4060, p < .001; multivariate kurtosis test statistic = 8.95, p < .001). Inference on structural and indirect paths therefore relied on bootstrap confidence intervals. Full-collinearity VIFs computed from construct mean scores ranged from 2.35 to 2.76. VIFs within each block of predictors ranged from 1.73 to 2.44, below the conservative threshold of 3.3 [66]. These diagnostics did not reveal an obvious single-factor or collinearity problem. They do not, however, rule out common method bias, which may still affect relationships estimated from cross-sectional self-reported data.

### Structural model and direct paths

All hypothesised direct paths were statistically significant; perceived explorability had the largest standardised path estimate for perceived cultural value (Table 9).

**Table 9:** Direct path results of the structural model.

Hypothesis	Path	beta	SE	t	p	95% BC CI	Result
H1a	IC - > PCV	0.202	0.065	3.078	0.002	[0.073, 0.333]	Supported
H1b	VA - > PCV	0.190	0.064	2.975	0.003	[0.060, 0.310]	Supported
H1c	PE - > PCV	0.364	0.081	4.517	<0.001	[0.207, 0.523]	Supported
H1d	PA - > PCV	0.183	0.075	2.435	0.015	[0.031, 0.329]	Supported
H2	PA - > CI	0.381	0.084	4.545	<0.001	[0.210, 0.539]	Supported
H3	PCV - > CI	0.505	0.079	6.376	<0.001	[0.347, 0.659]	Supported
H4	PCV - > CCEI	0.442	0.096	4.612	<0.001	[0.234, 0.618]	Supported
H5	CI - > CCEI	0.396	0.090	4.415	<0.001	[0.227, 0.575]	Supported

Note. Standardised coefficients. SE = bootstrap standard error; BC CI = bias-corrected bootstrap confidence interval (5,000 resamples).

The structural model explained 71.0% of the variance in PCV, 68.3% in CI, and 62.7% in CCEI.

### Indirect relationships and serial mediation paths

The bootstrap confidence intervals supported both value-mediated paths and serial value-identity paths from interpretive conditions to sustained engagement intention, together with an additional identity-mediated path from perceived authenticity (Table 10).

**Table 10:** Indirect effects and serial mediation results.

Hypothesis	Indirect path	Estimate	SE	t	p	95% BC CI	Result
H6a1	IC - > PCV - > CCEI	0.089	0.036	2.504	0.012	[0.029, 0.169]	Supported
H6a2	VA - > PCV - > CCEI	0.084	0.036	2.349	0.019	[0.023, 0.165]	Supported
H6a3	PE - > PCV - > CCEI	0.161	0.045	3.596	<0.001	[0.086, 0.261]	Supported
H6a4	PA - > PCV - > CCEI	0.081	0.043	1.889	0.059	[0.010, 0.174]	Supported†
H6b	PCV - > CI - > CCEI	0.200	0.044	4.592	<0.001	[0.129, 0.303]	Supported
Additional‡	PA - > CI - > CCEI	0.151	0.058	2.614	0.009	[0.054, 0.283]	Supported
H6c1	IC - > PCV - > CI - > CCEI	0.040	0.016	2.537	0.011	[0.016, 0.082]	Supported
H6c2	VA - > PCV - > CI - > CCEI	0.038	0.016	2.415	0.016	[0.014, 0.078]	Supported
H6c3	PE - > PCV - > CI - > CCEI	0.073	0.025	2.958	0.003	[0.036, 0.138]	Supported
H6c4	PA - > PCV - > CI - > CCEI	0.037	0.014	2.573	0.010	[0.013, 0.072]	Supported

Note. Standardised indirect effects. SE = bootstrap standard error; BC CI = bias-corrected bootstrap confidence interval (5,000 resamples). An indirect association was considered supported when the 95% BC CI excluded zero. The t statistic is the absolute estimate divided by its bootstrap SE; p values are the corresponding two-sided test results reported by SmartPLS. † For H6a4, the t-based p value was.059, whereas the 95% percentile CI [0.012, 0.179] and the 95% BC CI [0.010, 0.174] both excluded zero. Support for this path is based on the stated BC-CI criterion, although the t-based test did not reach the.05 level. ‡ The PA - > CI - > CCEI path was not separately hypothesised; it is reported for completeness because perceived authenticity has a direct path to cultural identity.

### fsQCA calibration and necessity analysis

The fsQCA calibration converted the six conditions and sustained cultural engagement intention into fuzzy sets (anchor values in S1 Appendix). Necessity analysis identified no individual condition or its absence as necessary for either high or low sustained cultural engagement intention at the 0.90 consistency threshold (Table 11).

**Table 11:** Necessity analysis for high/low sustained cultural engagement intention.

Condition	High CCEI consistency	High CCEI coverage	Low CCEI consistency	Low CCEI coverage
IC	0.797	0.805	0.507	0.523
~IC	0.528	0.512	0.811	0.803
VA	0.778	0.797	0.518	0.541
~VA	0.552	0.529	0.806	0.788
PE	0.809	0.800	0.527	0.532
~PE	0.526	0.522	0.801	0.811
PA	0.814	0.814	0.535	0.546
~PA	0.546	0.535	0.818	0.818
PCV	0.817	0.811	0.528	0.535
~PCV	0.531	0.525	0.813	0.819
CI	0.820	0.803	0.543	0.543
~CI	0.534	0.534	0.804	0.820

### Sufficient condition configuration analysis

The six conditions can form 64 logical configurations. Of these, 62 contained at least one case, and 29 met the frequency threshold of 3. These rows covered 300 of the 350 cases. Because 26 of the 29 rows exceeded the raw-consistency threshold of 0.80 for both high and low engagement, PRI screening was needed to separate the outcomes. Ten rows met both the raw-consistency (≥ 0.80) and PRI (≥ 0.65) criteria for high engagement. Ten did so for low engagement. No row met both criteria for both outcomes. The intermediate solution was identical to the complex solution for both outcomes. It yielded four configurations for high engagement and four for low engagement (Table 12; truth tables of all observed configurations in S1 Appendix).

**Table 12:** Sufficient condition configurations for high/low sustained cultural engagement intention.

Configuration	IC	VA	PE	PA	PCV	CI	Consistency	Raw coverage	Unique coverage	PRI
H1	●	●	○	●			0.930	0.586	0.050	0.858
H2	○			●	●	●	0.929	0.610	0.033	0.859
H3			●	●	●	●	0.923	0.622	0.046	0.848
H4	○	○	●		●	●	0.942	0.564	0.028	0.880
L1			⊗	⊗	⊕	⊗	0.944	0.615	0.033	0.888
L2	⊗		⊗	⊗	⊕		0.939	0.622	0.039	0.879
L3	⊗	⊗		⊕		⊗	0.938	0.606	0.055	0.877
L4	⊗	⊗	⊕		⊕	⊗	0.952	0.577	0.026	0.902
High-engagement solution							0.895	0.734	–	–
Low-engagement solution							0.907	0.736	–	–

Note: ● indicates core condition presence; ○ indicates peripheral condition presence; ⊗ indicates core condition absence; ⊕ indicates peripheral condition absence; a blank indicates that the condition was not specified in the configuration. Solution-level values refer to the intermediate solution (frequency threshold = 3; raw consistency ≥ 0.80; PRI ≥ 0.65; crossover memberships of 0.500 set to 0.501). Parsimonious solutions: high engagement, IC*VA*PA + PE*PCV*CI + PA*PCV*CI; low engagement, ~ IC* ~ VA* ~ CI + ~PE* ~ PA* ~ CI + ~IC* ~ PE* ~ PA.

All retained configurations had raw consistency above 0.92 and PRI consistency between 0.85 and 0.90, indicating limited overlap with the negation of the corresponding outcome.

For high engagement, H1 combines information clarity, visual aesthetics and perceived authenticity as core conditions. Perceived explorability is peripheral. H2 and H3 combine perceived authenticity, perceived cultural value and cultural identity. Information clarity supplements H2, whereas perceived explorability supplements H3. H4 combines perceived explorability, perceived cultural value and cultural identity as core conditions. Information clarity and visual aesthetics are peripheral, and perceived authenticity is not specified. Perceived cultural value and cultural identity appeared together in three of the four high-engagement configurations. Perceived authenticity also appeared in three. All four low-engagement configurations consisted of absent conditions. The L1 core comprised the absence of perceived explorability, perceived authenticity and cultural identity. L2 comprised the absence of information clarity, perceived explorability and perceived authenticity. L3 and L4 comprised the absence of information clarity, visual aesthetics and cultural identity. The absence of perceived cultural value or other conditions served as peripheral elements. The low-engagement configurations were not simple negations of the high-engagement configurations, indicating configurational asymmetry.

The robustness checks showed the same pattern. Raising the PRI threshold to 0.70 reduced solution coverage (high: 0.706; low: 0.705) and increased solution consistency (high: 0.907; low: 0.913). The resulting configurations were longer, but no condition changed direction. Unadjusted 0.500 memberships yielded similar solutions (S1 Appendix).

## Discussion

### Discussion of main findings

The findings associate perceived cultural value with users’ evaluations of the interpretive qualities of the Twenty-Four Solar Terms stimulus. Respondents evaluated the materials more positively when they could understand them, explore them and connect them with their own lives. This result is consistent with recent digital heritage research. That work shows that perceived value and engagement depend on how cultural information, experiential cues and contextual credibility are organised within digital experiences [71–73].

Perceived explorability had the largest standardised path estimate for perceived cultural value among the four interpretive conditions. This numerical ordering does not establish statistically significant differences between the path coefficients. This result extends heritage-interpretation and experience-design research, which emphasises users’ ability to organise, connect and further explore cultural information rather than merely receive it [31,36]. For living heritage design, this pattern highlights opportunities to connect cultural symbols with their associated meanings. Solar Terms names can lead to phenology, phenology to diet and bodily rhythms, and bodily experience to family memory, local customs and contemporary lifestyles. Explorability, as measured here, concerns perceived opportunities to discover connections among cultural meanings. An explorable presentation makes relationships among cultural meanings visible and leaves them open to further questioning.

The authenticity result adds a second explanation. In the CB-SEM model, perceived authenticity was positively associated with perceived cultural value and directly associated with cultural identity. This result is consistent with research that treats authenticity as a contextual and relational judgement rather than as the literal reproduction of an unchanged past [23,37]. Authenticity therefore functions as cultural trust. Users judge whether the digital expression follows the basic logic of Solar Terms culture and whether its phenology, diet, bodily experience and folk memories have been assembled coherently.

The fsQCA results add a configurational perspective. Perceived authenticity was a core condition in three of the four high-engagement configurations, but it was not necessary. In H4, high engagement was associated with perceived explorability, perceived cultural value and cultural identity, while information clarity and visual aesthetics were peripheral and authenticity was unspecified. CB-SEM identifies a positive sample-level association, whereas fsQCA shows that this association operates within different combinations of conditions [28,74]. Authenticity therefore matters, but engagement does not depend on it alone. It also depends on whether users can find understandable and accessible life meaning in the content.

The low-engagement configurations reinforce this point. Low engagement was not the mirror image of high engagement. It was associated with the joint absence of several conditions, most often low information clarity, low perceived explorability, low perceived authenticity and low cultural identity. This pattern is consistent with a broken interpretive chain. When information is not clearly organised, the content offers little room for further exploration, authenticity cues are weak and identity connection is absent. Respondents then reported little intention to continue engaging with digital Solar Terms content.

Together, the CB-SEM and fsQCA findings support a relational view of digital heritage evaluation. CB-SEM identified sample-level associations and indirect relationships. FsQCA showed that similar engagement outcomes could be associated with different combinations of interpretive and psychological conditions. This pattern is consistent with equifinality and configurational asymmetry.

### Research implications

Theoretically, the study examines perceived interpretive qualities in digital heritage evaluation. Within the S-O-R model, the stimulus was specified as users’ evaluations of interpretive qualities rather than media features. These qualities were associated with cultural value, cultural identity and sustained engagement. Explorability had the largest standardised path estimate for cultural value. Among the four interpretive conditions, authenticity was the only one specified as a direct predictor of cultural identity in the proposed model; direct paths from the other three conditions to identity were not tested. The findings suggest that the organisation and perceived credibility of cultural relationships are relevant to users’ evaluations of digital living heritage.

Methodologically, digital cultural translation links public digital discourse, thematic analysis, design mapping, expert review and user evaluation in one process. This differs from studies that analyse texts only or test users’ responses to existing digital products [12,73,75]. Combining CB-SEM with fsQCA further shows that high engagement can arise from different combinations of conditions. Low engagement is not the mirror image of high engagement. This distinction helps explain why information-rich and visually refined heritage projects may still fail to sustain engagement.

In practice, designers can identify themes that users recognise in public digital discourse. They can use expert review to check how those themes are translated into visual and content structures. User evaluation can then test whether the results are clear, explorable and credible. This process can reduce two common risks in traditional-culture digitisation: using traditional symbols without explaining their cultural relationships, and pursuing novel forms while weakening cultural context [71].

For the Twenty-Four Solar Terms, digital design should go beyond names, illustrations, colours and seasonal labels. It should build a continuous interpretive path around “time–nature–body–family–everyday life”. This path can move from phenological change to agricultural memory, from dietary habits to bodily perception, and from family scenes to contemporary life practice.

## Conclusion

We examined how public digital discourse on the Twenty-Four Solar Terms can be translated into digital interpretive material and how users evaluate the result. Six cultural themes identified in public discourse were translated into a five-page storyboard through theme-design mapping and expert review. Information clarity, visual aesthetics, perceived explorability and perceived authenticity were positively associated with perceived cultural value. Explorability had the largest standardised path estimate for cultural value, and perceived authenticity was also associated with cultural identity. Perceived cultural value and cultural identity linked these conditions to sustained cultural engagement intention through mediating and serial pathways. FsQCA identified four configurations each for high and low engagement, and the two sets were asymmetric rather than mirror images.

## Limitations and future research

The study has four limitations. First, the survey data are cross-sectional. The CB-SEM and fsQCA results therefore describe associations and configurations rather than causal effects. Experimental and longitudinal designs could test whether these patterns persist and lead to actual learning, sharing or participation. Second, the five-page static storyboard ensured consistent exposure. Future studies could examine the model with interactive prototypes, digital exhibitions, virtual or augmented reality environments and behavioural log data. Third, the corpus drew mainly on public web pages, and the sample was relatively young. Adding social-media, short-video, interview and field materials could broaden the corpus. Comparisons across age, region and cultural experience could also test transportability. Finally, sustained cultural engagement intention is a proximal indicator of cultural vitality. Combining it with platform behavioural data would show how digital contact becomes sustained cultural practice.

## Supporting information

S1 Data Anonymised item-level survey dataset. The file contains the final 350 respondent-level records used in the CB-SEM and fsQCA analyses, with direct identifiers and submission metadata removed or anonymised.
(XLSX)

S1 Appendix Supplementary methodological documentation. The file contains stimulus-development notes, the full questionnaire items, construct scoring, corpus retrieval strings and screening counts, the CB-SEM specification and diagnostics, and fsQCA calibration anchors, truth tables, solutions, and sensitivity analyses.
(DOCX)

S2 Code Corpus-processing workflow. The file documents text screening, Chinese word segmentation, keyword extraction, auxiliary topic-model inspection, and related corpus-processing procedures.
(ZIP)
