# Supplementary material for: From public digital discourse to sustained engagement: Digital cultural translation of the Twenty-Four Solar Terms
Source: PLoS One. 2026 Oct 27;21(10):e0361505. doi: 10.1371/journal.pone.0361505 (PMC13712605; doi:10.1371/journal.pone.0361505)
Supplement: Supplementary file 1 [file pone.0361505.s002.docx]

**S1 Appendix. Supplementary methodological documentation**

*From public digital discourse to sustained engagement: Digital cultural translation of the Twenty-Four Solar Terms*

Reference numbers in square brackets refer to the reference list of the main manuscript.

## **A.1 Purpose of this appendix**

This appendix documents the development of the storyboard stimulus used in the questionnaire, the full questionnaire items, the corpus retrieval strings and screening counts, the CB-SEM specification and diagnostics, and the fsQCA calibration, truth tables, solutions, and sensitivity analyses. The stimulus was not treated as a separate latent variable. Digital cultural translation served as a stimulus-development procedure linking public digital discourse, thematic analysis, expert-assisted design mapping, and user evaluation.

The terminology follows the main manuscript: information clarity (IC), visual aesthetics (VA), perceived explorability (PE), perceived authenticity (PA), perceived cultural value (PCV), cultural identity (CI), and sustained cultural engagement intention (CCEI).

## **A.2 Selection of Solar Terms cases**

The storyboard focused on the Four Beginnings: Beginning of Spring (Lichun), Beginning of Summer (Lixia), Beginning of Autumn (Liqiu), and Beginning of Winter (Lidong). These cases mark the entry points of the four seasonal phases and allow the Twenty-Four Solar Terms to be presented as an annual calendrical system within a limited five-page stimulus.

The Four Beginnings served three purposes. First, they provide seasonal representativeness: viewers encounter a complete cycle of spring, summer, autumn, and winter rather than isolated Solar Terms. Second, they provide cognitive accessibility: the four seasonal beginnings are widely recognisable, reducing the likelihood that unfamiliarity with individual Solar Terms would dominate respondents' evaluations. Third, they provide content sufficiency: each is associated with folk customs, dietary practices, bodily routines, phenological signs, and family or everyday memory.

## **A.3 Corpus-derived cultural themes and stimulus mapping**

The six cultural theme categories identified in the main study were not statistical variables. They supplied the cultural evidence base for constructing the stimulus. The research team developed these categories through joint reading, grouping of recurring expressions, and discussion of shared meanings.

**Table A1. Cultural theme categories and their use in the storyboard stimulus.**

| **Cultural theme category** | **Cultural basis in public discourse** | **Use in storyboard stimulus** |
| --- | --- | --- |
| Seasonal diet and bodily care | Dietary customs, nourishment, bodily adjustment, family health care | Food-custom module, bodily-rhythm cues, seasonal care information |
| Temporal order and heritage knowledge | Annual cycle, Solar Terms sequence, time knowledge, agricultural timing | Opening overview page, annual-cycle navigation, structured knowledge explanation |
| Natural phenology and seasonal perception | Weather signs, plants, dew, frost, snow, and sensory seasonal change | Phenological cue cards, environmental imagery, seasonal transition prompts |
| Folk customs and ritual life | Welcoming spring, biting spring, seasonal rituals, family customs, ritual memory | Folk-custom section, family-memory prompts, everyday practice examples |
| Aesthetic imagery and digital creative expression | Seasonal colours, poetic atmosphere, illustration, contemporary visual communication | Visual style, illustration language, colour system, interface atmosphere |
| Agricultural production and everyday practice | Agricultural rhythms, crop growth, rural memory, seasonal labour | Agricultural memory content, everyday practice cases, seasonal labour references |

## **A.4 Visual design system**

The stimulus used a contemporary, interface-oriented visual system while retaining culturally grounded cues. The opening page established the Twenty-Four Solar Terms as a calendrical-cultural system; subsequent pages used seasonal colour, grid-based layout, section headings, and content zoning to support readability.

**Table A2. Visual design elements.**

| **Design element** | **Design principle** | **Main evaluative construct supported** |
| --- | --- | --- |
| Seasonal colour coding | Distinct chromatic identity for each seasonal node | Visual aesthetics; seasonal differentiation |
| Opening cultural image | Traditional visual cue concentrated at the overview entry point | Perceived authenticity; visual anchoring |
| Column-based grid and content zoning | Clear division of knowledge, customs, everyday practice, and cultural meaning | Information clarity |
| Layered page structure | Progressive movement from basic knowledge to cultural meaning | Perceived explorability |
| Two-level typography hierarchy | Readable body text and more distinctive section headings | Information clarity; visual hierarchy |

## **A.5 Content architecture and perceived explorability**

The stimulus contained five thematic pages rather than one page for each Solar Term. Its page structure is identical to that reported in the main manuscript.

**Table A3. Page structure of the five-page storyboard.**

| **Page** | **Content focus** | **Main theme category** |
| --- | --- | --- |
| Page 1 | The Twenty-Four Solar Terms as a calendrical and cultural system; annual-cycle navigation | Temporal order and heritage knowledge |
| Page 2 | Phenological symbols, seasonal cue cards, and agricultural memory | Natural phenology and seasonal perception; agricultural production and everyday practice |
| Page 3 | The Four Beginnings connected with dietary customs and bodily rhythms | Seasonal diet and bodily care |
| Page 4 | Folk practices, family memory, and everyday life | Folk customs and ritual life; agricultural production and everyday practice |
| Page 5 | Poetic imagery and participatory communication translated into contemporary interface logic, with prompts to continue exploring | Aesthetic imagery and digital creative expression |

The sequence moves from the calendrical system (Page 1) to natural signs (Page 2), bodily and dietary practice (Page 3), family and everyday memory (Page 4), and contemporary expression and further exploration (Page 5). In the static storyboard, perceived explorability was operationalised through layered content, overview-to-detail progression, and cues for further cultural interpretation rather than clickable navigation or interactive functions.

## **A.6 Operationalisation of interpretive conditions**

**Table A4. Correspondence between stimulus features and measured constructs.**

| **Construct** | **Stimulus or measurement basis** | **Role in the evaluation model** |
| --- | --- | --- |
| Information clarity (IC) | Clear section headings, concise explanations, annual-cycle structure, readable information zoning | Whether users could understand the Solar Terms knowledge and page organisation |
| Visual aesthetics (VA) | Seasonal colour coding, contemporary layout, visual hierarchy, culturally coherent atmosphere | Whether users perceived the visual presentation as pleasant and thematically appropriate |
| Perceived explorability (PE) | Overview-to-detail progression across pages; links among phenology, diet, body, family, and everyday life; prompts on Page 5 | Whether users felt that the content had further layers and connections to explore |
| Perceived authenticity (PA) | Corpus-based cultural content, folk customs, everyday-life context, traditional visual cue at the entry point | Whether users perceived the stimulus as culturally credible and connected to living practice |
| Perceived cultural value (PCV) | Measured as an Organism-level evaluation after viewing | Whether users judged the digital material as culturally meaningful today |
| Cultural identity (CI) | Measured as an Organism-level response after viewing | Whether users felt culturally connected to the Solar Terms after viewing |
| Sustained cultural engagement intention (CCEI) | Measured as a Response-level intention after viewing | Whether users were willing to continue learning, browsing, sharing, or engaging with Solar Terms content |

## **A.7 Expert-assisted review**

After completing the initial theme-design mapping, the research team asked nine reviewers with design-related academic training or research experience to assess the correspondence between the cultural themes and storyboard components. Their backgrounds covered cultural creative design, visual and interaction design, digital heritage communication, and design research methods. The reviewers rated and commented on whether the content structure, cultural examples, visual hierarchy, and page sequence adequately represented the themes identified from the corpus.

Reviewer feedback led to targeted revisions in the knowledge structure, bodily-practice cues, phenological presentation, ritual and family-memory examples, visual imagery, and prompts for continued exploration (main manuscript, Table 4). We completed these revisions before the formal survey. The expert review was a consultation on the design materials: no personal or identifying information was collected from the reviewers, and their comments were used only to revise the stimulus rather than analysed as research data.

## **A.8 Full questionnaire items**

The formal questionnaire was administered through the Wenjuanxing online survey platform. It contained screening and background questions, an attention-check item, and seven reflective construct scales. Construct items are English translations of the Chinese wording and were rated from 1 (strongly disagree) to 7 (strongly agree).

**Table A5. Screening, background, and attention-check items.**

| **Item** | **Block** | **Question wording or function** | **Response format** |
| --- | --- | --- | --- |
| Q1 | Adult eligibility | Are you 18 years old or above? | Screening item |
| Q2 | Stimulus-viewing eligibility | Have you fully viewed the five-page digital exhibition sample? | Screening item |
| Q3 | Prior Solar Terms knowledge | Before this survey, how familiar were you with the Twenty-Four Solar Terms? | Ordinal |
| Q4 | Digital cultural-content contact | How often do you usually encounter digital content about traditional culture? | Ordinal |
| Q5 | Digital exhibition experience | In the past year, have you viewed digital exhibitions, virtual museums, or online cultural-display pages? | Ordinal |
| Q6 | Most impressive content | After viewing the Solar Terms digital exhibition sample, which type of content impressed you most? | Categorical |
| Q10 | Attention check | To ensure questionnaire quality, please select ‘5 somewhat agree’ for this item. | Attention check |
| Q15–Q19 | Demographics | Gender, age group, highest education, occupation, and region of residence | Categorical |

**Table A6. Construct items.**

| **Construct (sources)** | **Code** | **Item wording** |
| --- | --- | --- |
| Information clarity [10,19,31-32] | IC1 | I could quickly identify the core theme of this set of digital content. |
|  | IC2 | The information conveyed by this set of digital content was clear. |
|  | IC3 | I could easily understand the knowledge of the Twenty-Four Solar Terms presented in the content. |
|  | IC4 | This set of content was clearly organised in terms of information structure. |
| Visual aesthetics [10,19,33,48] | VA1 | The overall visual presentation of this set of digital content felt comfortable to me. |
|  | VA2 | The colours, layout, and overall style of this content showed good aesthetic coordination. |
|  | VA3 | The visual presentation of this content was consistent with the theme of the Twenty-Four Solar Terms. |
|  | VA4 | This content left me with a positive aesthetic impression. |
| Perceived explorability [34-36] | PE1 | This content presented knowledge cues about the culture of the Twenty-Four Solar Terms that were worth further exploration. |
|  | PE2 | This content provided cues for me to further explore the culture of the Twenty-Four Solar Terms. |
|  | PE3 | This content made me feel that there was more to discover behind the Twenty-Four Solar Terms. |
|  | PE4 | This content helped me see richer connections among seasons, everyday life, and traditional culture. |
| Perceived authenticity [23,37,40-41] | PA1 | I think this set of digital content authentically preserved the core characteristics of the Twenty-Four Solar Terms. |
|  | PA2 | This content presented not only surface elements but also authentic cultural meanings. |
|  | PA3 | This content allowed me to sense the cultural background and everyday-life context behind the Twenty-Four Solar Terms. |
|  | PA4 | Even though it was presented digitally, this content still maintained the cultural spirit of the Twenty-Four Solar Terms. |
| Perceived cultural value [9,12-13] | PCV1 | This digital content made me realise that the Twenty-Four Solar Terms still have important cultural meaning today. |
|  | PCV2 | This content not only conveyed Solar Terms knowledge but also allowed me to perceive its value for contemporary life. |
|  | PCV3 | This content helped me understand the connection between the Twenty-Four Solar Terms and modern life. |
|  | PCV4 | This content made me further recognise that the culture of the Twenty-Four Solar Terms deserves sustained transmission. |
| Cultural identity [9,38-39] | CI1 | After viewing this content, I felt closer to the culture of the Twenty-Four Solar Terms. |
|  | CI2 | This content strengthened my sense of identification with Chinese traditional seasonal culture. |
|  | CI3 | I am willing to regard the Twenty-Four Solar Terms as a tradition related to my life or cultural background. |
|  | CI4 | This content made me feel connected to this seasonal culture. |
| Sustained cultural engagement intention [9-10,12-13,19] | CCEI1 | After viewing this content, I would like to continue learning about knowledge, customs, or digital content related to the Twenty-Four Solar Terms. |
|  | CCEI2 | In the future, I would like to continue following digital exhibitions, online displays, or cultural activities related to the Twenty-Four Solar Terms. |
|  | CCEI3 | I would be willing to share or recommend digital content related to the Twenty-Four Solar Terms to others. |
|  | CCEI4 | I would be willing to try to understand or use lifestyle knowledge related to the Twenty-Four Solar Terms in daily life. |

## **A.9 Corpus retrieval strings and screening**

Publicly visible Chinese-language materials were retrieved in March 2026 using the 21 retrieval strings below. The strings are reported in the original Chinese with English glosses; “Four Beginnings” stands for 立春 立夏 立秋 立冬. Counts refer to records retained in the final 1,101-record corpus. Item-level retrieval timestamps were not retained. The field publish_date_or_access_date records the dataset curation date (June 1, 2026), not the publication date or retrieval date of each source.

**Table A7. Retrieval strings and retained records.**

| **No.** | **Retrieval string (Chinese)** | **English gloss** | **Retained** |
| --- | --- | --- | --- |
| 1 | 立夏 习俗 物候 饮食 养生 | Lixia; customs; phenology; diet; health preservation | 114 |
| 2 | 立春 立夏 立秋 立冬 饮食 养生 人民网 新华网 央视网 | Four Beginnings; diet; health preservation; People.cn; Xinhuanet; CCTV.com | 106 |
| 3 | 立春 习俗 物候 文化 养生 | Lichun; customs; phenology; culture; health preservation | 85 |
| 4 | 立春 立夏 立秋 立冬 养生 健康 科普 官方 卫健委 | Four Beginnings; health preservation; health; popular science; official; health commission | 76 |
| 5 | 二十四节气 非遗 文化传承 学术论文 摘要 | Twenty-Four Solar Terms; ICH; cultural transmission; academic paper; abstract | 75 |
| 6 | 二十四节气 吧 贴吧 立春 立夏 立秋 立冬 习俗 | Twenty-Four Solar Terms; Baidu Tieba forum; Four Beginnings; customs | 74 |
| 7 | 立秋 习俗 物候 饮食 养生 | Liqiu; customs; phenology; diet; health preservation | 66 |
| 8 | 二十四节气 中国非物质文化遗产网 立春 立夏 立秋 立冬 | Twenty-Four Solar Terms; China ICH website; Four Beginnings | 61 |
| 9 | 二十四节气 官方 文化 非遗 公共文化 立春 立夏 立秋 立冬 | Twenty-Four Solar Terms; official; culture; ICH; public culture; Four Beginnings | 56 |
| 10 | 立冬 习俗 物候 饮食 养生 | Lidong; customs; phenology; diet; health preservation | 55 |
| 11 | 二十四节气 文创 视觉设计 数字展览 学术 研究 | Twenty-Four Solar Terms; cultural-creative products; visual design; digital exhibition; academic; research | 48 |
| 12 | 二十四节气 全国农业展览馆 中国农业博物馆 立春 立夏 立秋 立冬 | Twenty-Four Solar Terms; National Agricultural Exhibition Center; China Agricultural Museum; Four Beginnings | 47 |
| 13 | 二十四节气 UNESCO 非物质文化遗产 中文 | Twenty-Four Solar Terms; UNESCO; ICH; Chinese | 46 |
| 14 | 二十四节气 小红书 抖音 微博 B站 文案 立春 立夏 立秋 立冬 | Twenty-Four Solar Terms; Xiaohongshu; Douyin; Weibo; Bilibili; copywriting; Four Beginnings | 43 |
| 15 | 二十四节气 用户讨论 论坛 习俗 立春 立夏 立秋 立冬 | Twenty-Four Solar Terms; user discussion; forum; customs; Four Beginnings | 34 |
| 16 | 立春 立夏 立秋 立冬 文创 商品 设计 海报 淘宝 京东 | Four Beginnings; cultural-creative products; merchandise; design; poster; Taobao; JD.com | 31 |
| 17 | 二十四节气 文创 淘宝 天猫 京东 拼多多 商品 文案 | Twenty-Four Solar Terms; cultural-creative products; Taobao; Tmall; JD.com; Pinduoduo; merchandise; copywriting | 28 |
| 18 | 二十四节气 文化传播 数字化 设计 学术论文 摘要 | Twenty-Four Solar Terms; cultural communication; digitisation; design; academic paper; abstract | 26 |
| 19 | 二十四节气 百度百科 立春 立夏 立秋 立冬 | Twenty-Four Solar Terms; Baidu Baike; Four Beginnings | 11 |
| 20 | 二十四节气 | Twenty-Four Solar Terms | 10 |
| 21 | 二十四节气 文化 官方 习俗 非遗 | Twenty-Four Solar Terms; culture; official; customs; ICH | 9 |
|  | Total |  | 1101 |

**Table A8. Screening counts (implemented in S2 Code, clean_corpus.py).**

| **Step** | **Records** |
| --- | --- |
| Raw candidate text units | 3,433 |
| Excluded: not related to the Solar Terms (no mention of the Solar Terms or of a named Solar Term) | 2,202 |
| Excluded: advertising or site noise | 80 |
| Excluded: exact duplicate after cleaning and whitespace removal | 50 |
| Excluded: too short (< 10 Chinese characters) or non-Chinese | 0 |
| Retained, cleaned, and anonymised text units | 1,101 |

Lexical analyses (S2 Code, analyse_themes.py) first applied jieba segmentation to the cleaned_text field, then removed tokens shorter than two characters, stop words and tokens containing no Chinese characters. The script excluded documents with fewer than three remaining tokens. Re-execution on the retained 1,101-record Chinese corpus excluded 12 documents and retained 1,089 for word frequencies, TF-IDF (scikit-learn TfidfVectorizer, mean scores for the whole computational corpus and each Solar Terms group) and LDA (scikit-learn LatentDirichletAllocation, batch learning, random state 42, 4–8 topics, 12 top words). The 1,101 records remained available for collaborative thematic reading. The exact Python and package versions used in the original analysis were not recorded. During this revision, the supplied workflow was successfully re-executed with Python 3.12.14, pandas 2.2.3, jieba 0.42.1, scikit-learn 1.8.0, NumPy 2.3.5 and SciPy 1.17.0. These versions describe the verified re-execution environment, not the original analysis environment. The dependency list, re-execution command and document-count check are provided in S2 Code.

## **A.10 CB-SEM specification and diagnostics**

Software: SmartPLS 4.1.0.9, CB-SEM algorithm; maximum-likelihood estimation; no mean structure; stopping criteria 10⁻⁹ (function value) and 10⁻⁶ (gradient). Each latent variable was scaled by fixing its first loading to 1. Bootstrapping used 5,000 nonparametric resamples; the manuscript reports standardised estimates with bootstrap standard errors and bias-corrected confidence intervals.

Measurement model: IC =~ IC1 + IC2 + IC3 + IC4; VA =~ VA1 + VA2 + VA3 + VA4; PE =~ PE1 + PE2 + PE3 + PE4; PA =~ PA1 + PA2 + PA3 + PA4; PCV =~ PCV1 + PCV2 + PCV3 + PCV4; CI =~ CI1 + CI2 + CI3 + CI4; CCEI =~ CCEI1 + CCEI2 + CCEI3 + CCEI4.

Structural model: PCV ~ IC + VA + PE + PA; CI ~ PA + PCV; CCEI ~ PCV + CI; the four exogenous constructs were allowed to covary. The structural model had 70 free parameters and 336 degrees of freedom; the seven-factor CFA had 77 free parameters and 329 degrees of freedom; the one-factor CFA had 56 free parameters and 350 degrees of freedom.

**Table A9. Item-level distribution diagnostics (N = 350).**

| **Item** | **Mean** | **SD** | **Skewness** | **Excess kurtosis** |
| --- | --- | --- | --- | --- |
| IC1 | 3.886 | 1.289 | -0.141 | -0.324 |
| IC2 | 3.886 | 1.356 | -0.020 | -0.562 |
| IC3 | 3.917 | 1.307 | -0.024 | -0.268 |
| IC4 | 3.900 | 1.300 | 0.021 | -0.525 |
| VA1 | 4.169 | 1.250 | -0.110 | -0.206 |
| VA2 | 4.131 | 1.305 | -0.160 | -0.354 |
| VA3 | 4.057 | 1.292 | 0.037 | -0.352 |
| VA4 | 4.126 | 1.318 | -0.060 | -0.278 |
| PE1 | 3.957 | 1.301 | -0.125 | -0.369 |
| PE2 | 3.960 | 1.321 | 0.096 | -0.383 |
| PE3 | 3.957 | 1.346 | -0.014 | -0.309 |
| PE4 | 3.951 | 1.307 | 0.021 | -0.241 |
| PA1 | 4.111 | 1.281 | -0.169 | -0.011 |
| PA2 | 4.120 | 1.268 | -0.134 | -0.211 |
| PA3 | 4.174 | 1.335 | -0.199 | -0.212 |
| PA4 | 4.131 | 1.276 | -0.306 | -0.258 |
| PCV1 | 4.174 | 1.303 | 0.002 | -0.438 |
| PCV2 | 4.123 | 1.250 | 0.102 | -0.265 |
| PCV3 | 4.091 | 1.286 | 0.251 | -0.310 |
| PCV4 | 4.166 | 1.230 | 0.099 | -0.538 |
| CI1 | 4.054 | 1.313 | 0.037 | -0.265 |
| CI2 | 4.020 | 1.361 | -0.043 | -0.205 |
| CI3 | 4.017 | 1.300 | -0.166 | -0.225 |
| CI4 | 4.000 | 1.271 | 0.008 | -0.160 |
| CCEI1 | 4.286 | 1.313 | -0.081 | -0.413 |
| CCEI2 | 4.214 | 1.370 | 0.039 | -0.360 |
| CCEI3 | 4.249 | 1.275 | -0.016 | -0.360 |
| CCEI4 | 4.189 | 1.330 | -0.085 | -0.324 |

Mardia's multivariate skewness: χ² = 4702.48, df = 4060, p < .001; Mardia's multivariate kurtosis test statistic = 8.95, p < .001 (SmartPLS output).

**Table A10. Variance inflation factors from construct mean scores.**

| **Construct** | **Full-collinearity VIF** | **Block VIF (dependent construct)** |
| --- | --- | --- |
| IC | 2.466 | 2.291 (PCV) |
| VA | 2.347 | 2.163 (PCV) |
| PE | 2.756 | 2.443 (PCV) |
| PA | 2.514 | 2.207 (PCV); 1.732 (CI) |
| PCV | 2.749 | 1.732 (CI); 1.975 (CCEI) |
| CI | 2.621 | 1.975 (CCEI) |
| CCEI | 2.519 | — |

## **A.11 fsQCA calibration, truth tables, and solutions**

Software: fsQCA 4.1. Construct scores were the means of the four items of each construct; no items were reverse-coded. Direct calibration used the 5th, 50th, and 95th sample percentiles as full non-membership, crossover, and full membership anchors. Memberships of exactly 0.500 were set to 0.501 in the main analysis. Necessity threshold: consistency ≥ 0.90. Sufficiency: frequency threshold 3; raw consistency ≥ 0.80; PRI ≥ 0.65 (0.70 in the robustness check). Rows meeting the raw-consistency threshold but not the PRI threshold were coded 0. Directional expectations for the intermediate solution: all six conditions present for high CCEI and absent for low CCEI.

**Table A11. Calibration anchors.**

| **Set** | **Full non-membership (5th)** | **Crossover (50th)** | **Full membership (95th)** | **Cases at 0.500 adjusted to 0.501** |
| --- | --- | --- | --- | --- |
| IC | 2.000 | 4.000 | 5.750 | 24 |
| VA | 2.250 | 4.250 | 6.000 | 28 |
| PE | 2.000 | 4.000 | 5.750 | 26 |
| PA | 2.113 | 4.250 | 6.000 | 39 |
| PCV | 2.250 | 4.125 | 6.000 | 0 |
| CI | 2.000 | 4.000 | 6.000 | 23 |
| CCEI | 2.250 | 4.250 | 6.250 | 22 |

**Table A12. Truth table of all observed configurations for high and low CCEI (crossover adjustment 0.501).**

| **IC** | **VA** | **PE** | **PA** | **PCV** | **CI** | **n** | **High raw cons.** | **High PRI** | **High out** | **Low raw cons.** | **Low PRI** | **Low out** |
| --- | --- | --- | --- | --- | --- | --- | --- | --- | --- | --- | --- | --- |
| 1 | 1 | 1 | 1 | 1 | 1 | 75 | 0.950 | 0.896 | 1 | 0.565 | 0.084 | 0 |
| 0 | 0 | 0 | 0 | 0 | 0 | 72 | 0.550 | 0.059 | 0 | 0.963 | 0.923 | 1 |
| 1 | 0 | 1 | 1 | 1 | 1 | 13 | 0.951 | 0.816 | 1 | 0.782 | 0.177 | 0 |
| 1 | 1 | 1 | 1 | 0 | 1 | 11 | 0.943 | 0.738 | 1 | 0.839 | 0.260 | 0 |
| 0 | 1 | 1 | 1 | 1 | 1 | 10 | 0.940 | 0.752 | 1 | 0.818 | 0.248 | 0 |
| 0 | 0 | 0 | 1 | 0 | 0 | 8 | 0.825 | 0.206 | 0 | 0.955 | 0.794 | 1 |
| 1 | 1 | 1 | 0 | 1 | 1 | 8 | 0.950 | 0.786 | 1 | 0.815 | 0.214 | 0 |
| 1 | 1 | 1 | 1 | 1 | 0 | 8 | 0.957 | 0.796 | 1 | 0.832 | 0.204 | 0 |
| 0 | 0 | 0 | 0 | 0 | 1 | 8 | 0.820 | 0.214 | 0 | 0.950 | 0.779 | 1 |
| 0 | 1 | 0 | 0 | 0 | 0 | 7 | 0.833 | 0.195 | 0 | 0.959 | 0.803 | 1 |
| 1 | 0 | 0 | 1 | 1 | 1 | 6 | 0.944 | 0.699 | 1 | 0.869 | 0.301 | 0 |
| 1 | 1 | 0 | 0 | 0 | 0 | 6 | 0.893 | 0.309 | 0 | 0.952 | 0.691 | 1 |
| 1 | 1 | 0 | 1 | 1 | 1 | 6 | 0.946 | 0.737 | 1 | 0.848 | 0.263 | 0 |
| 1 | 1 | 0 | 0 | 1 | 1 | 5 | 0.935 | 0.623 | 0 | 0.891 | 0.371 | 0 |
| 0 | 0 | 1 | 0 | 0 | 0 | 5 | 0.840 | 0.196 | 0 | 0.961 | 0.803 | 1 |
| 0 | 1 | 1 | 1 | 1 | 0 | 5 | 0.939 | 0.637 | 0 | 0.893 | 0.363 | 0 |
| 0 | 0 | 0 | 0 | 1 | 0 | 5 | 0.831 | 0.201 | 0 | 0.956 | 0.791 | 1 |
| 1 | 0 | 0 | 0 | 0 | 1 | 4 | 0.894 | 0.365 | 0 | 0.939 | 0.635 | 0 |
| 1 | 0 | 1 | 1 | 0 | 0 | 4 | 0.925 | 0.525 | 0 | 0.917 | 0.475 | 0 |
| 0 | 0 | 1 | 1 | 0 | 1 | 4 | 0.913 | 0.504 | 0 | 0.911 | 0.496 | 0 |
| 0 | 1 | 0 | 0 | 0 | 1 | 4 | 0.885 | 0.304 | 0 | 0.950 | 0.696 | 1 |
| 0 | 0 | 1 | 1 | 1 | 1 | 4 | 0.930 | 0.652 | 1 | 0.870 | 0.348 | 0 |
| 0 | 0 | 1 | 0 | 0 | 1 | 4 | 0.888 | 0.356 | 0 | 0.937 | 0.635 | 0 |
| 0 | 0 | 1 | 0 | 1 | 0 | 3 | 0.882 | 0.344 | 0 | 0.938 | 0.656 | 1 |
| 1 | 1 | 1 | 0 | 0 | 0 | 3 | 0.917 | 0.448 | 0 | 0.933 | 0.552 | 0 |
| 1 | 1 | 1 | 1 | 0 | 0 | 3 | 0.948 | 0.687 | 1 | 0.884 | 0.307 | 0 |
| 0 | 0 | 0 | 1 | 0 | 1 | 3 | 0.886 | 0.378 | 0 | 0.931 | 0.622 | 0 |
| 1 | 0 | 0 | 1 | 0 | 0 | 3 | 0.891 | 0.374 | 0 | 0.935 | 0.626 | 0 |
| 1 | 0 | 0 | 0 | 0 | 0 | 3 | 0.843 | 0.205 | 0 | 0.959 | 0.795 | 1 |
| 1 | 0 | 1 | 0 | 1 | 0 | 2 | 0.927 | 0.533 | R | 0.915 | 0.456 | R |
| 1 | 0 | 1 | 0 | 0 | 0 | 2 | 0.909 | 0.413 | R | 0.936 | 0.586 | R |
| 1 | 1 | 1 | 0 | 1 | 0 | 2 | 0.937 | 0.635 | R | 0.890 | 0.365 | R |
| 0 | 1 | 0 | 0 | 1 | 1 | 2 | 0.912 | 0.467 | R | 0.923 | 0.533 | R |
| 0 | 1 | 1 | 1 | 0 | 1 | 2 | 0.943 | 0.676 | R | 0.881 | 0.324 | R |
| 0 | 0 | 0 | 1 | 1 | 1 | 2 | 0.918 | 0.554 | R | 0.898 | 0.446 | R |
| 0 | 0 | 0 | 1 | 1 | 0 | 2 | 0.892 | 0.357 | R | 0.940 | 0.643 | R |
| 0 | 0 | 0 | 0 | 1 | 1 | 2 | 0.895 | 0.427 | R | 0.922 | 0.573 | R |
| 0 | 1 | 0 | 1 | 0 | 0 | 2 | 0.902 | 0.370 | R | 0.942 | 0.630 | R |
| 0 | 1 | 0 | 1 | 0 | 1 | 2 | 0.925 | 0.499 | R | 0.925 | 0.501 | R |
| 0 | 1 | 0 | 1 | 1 | 1 | 2 | 0.931 | 0.587 | R | 0.903 | 0.413 | R |
| 1 | 0 | 1 | 1 | 0 | 1 | 2 | 0.951 | 0.707 | R | 0.882 | 0.293 | R |
| 1 | 0 | 1 | 1 | 1 | 0 | 2 | 0.942 | 0.661 | R | 0.886 | 0.334 | R |
| 1 | 1 | 1 | 0 | 0 | 1 | 2 | 0.934 | 0.576 | R | 0.909 | 0.414 | R |
| 1 | 1 | 0 | 1 | 1 | 0 | 2 | 0.949 | 0.646 | R | 0.908 | 0.354 | R |
| 1 | 1 | 0 | 1 | 0 | 0 | 2 | 0.931 | 0.528 | R | 0.923 | 0.472 | R |
| 1 | 1 | 0 | 1 | 0 | 1 | 2 | 0.945 | 0.646 | R | 0.900 | 0.354 | R |
| 1 | 1 | 0 | 0 | 1 | 0 | 1 | 0.929 | 0.479 | R | 0.935 | 0.521 | R |
| 1 | 1 | 0 | 0 | 0 | 1 | 1 | 0.927 | 0.458 | R | 0.938 | 0.542 | R |
| 0 | 1 | 1 | 0 | 1 | 1 | 1 | 0.930 | 0.626 | R | 0.882 | 0.374 | R |
| 1 | 0 | 0 | 0 | 1 | 1 | 1 | 0.924 | 0.564 | R | 0.902 | 0.436 | R |
| 1 | 0 | 0 | 1 | 1 | 0 | 1 | 0.928 | 0.519 | R | 0.922 | 0.481 | R |
| 1 | 0 | 0 | 1 | 0 | 1 | 1 | 0.932 | 0.548 | R | 0.918 | 0.452 | R |
| 1 | 0 | 1 | 0 | 1 | 1 | 1 | 0.941 | 0.678 | R | 0.875 | 0.313 | R |
| 1 | 0 | 1 | 0 | 0 | 1 | 1 | 0.932 | 0.514 | R | 0.928 | 0.486 | R |
| 0 | 1 | 1 | 1 | 0 | 0 | 1 | 0.933 | 0.569 | R | 0.911 | 0.431 | R |
| 0 | 1 | 1 | 0 | 0 | 1 | 1 | 0.915 | 0.457 | R | 0.928 | 0.543 | R |
| 0 | 0 | 1 | 1 | 1 | 0 | 1 | 0.915 | 0.483 | R | 0.921 | 0.517 | R |
| 0 | 1 | 0 | 0 | 1 | 0 | 1 | 0.896 | 0.325 | R | 0.950 | 0.675 | R |
| 0 | 1 | 1 | 0 | 0 | 0 | 1 | 0.885 | 0.303 | R | 0.950 | 0.697 | R |
| 0 | 1 | 0 | 1 | 1 | 0 | 1 | 0.923 | 0.481 | R | 0.929 | 0.519 | R |
| 0 | 0 | 1 | 1 | 0 | 0 | 1 | 0.904 | 0.383 | R | 0.939 | 0.604 | R |
| 0 | 0 | 1 | 0 | 1 | 1 | 1 | 0.909 | 0.508 | R | 0.906 | 0.492 | R |

Note. 1 = membership > 0.5 in the condition; n = number of cases with membership > 0.5 in the row. Out: 1 = coded sufficient (raw consistency ≥ 0.80 and PRI ≥ 0.65); 0 = not sufficient; R = logical remainder (n < 3). Of the 64 logically possible rows, 62 contained at least one case and 2 contained none (not shown). The 29 rows with n ≥ 3 covered 300 of 350 cases; 26 of them exceeded raw consistency 0.80 for both outcomes, and no row was coded 1 for both outcomes.

**Table A13. Solutions (crossover adjustment 0.501; PRI ≥ 0.65).**

| **Outcome** | **Solution** | **Configuration** | **Raw coverage** | **Unique coverage** | **Consistency** |
| --- | --- | --- | --- | --- | --- |
| High CCEI | Complex = intermediate | IC*VA*PE*PA | 0.586 | 0.050 | 0.930 |
|  |  | IC*PA*PCV*CI | 0.610 | 0.033 | 0.929 |
|  |  | PE*PA*PCV*CI | 0.622 | 0.046 | 0.923 |
|  |  | IC*VA*PE*PCV*CI | 0.564 | 0.028 | 0.942 |
|  |  | Solution | 0.734 | — | 0.895 |
|  | Parsimonious | IC*VA*PA + PE*PCV*CI + PA*PCV*CI | 0.772 | — | 0.878 |
| Low CCEI | Complex = intermediate | ~PE*~PA*~PCV*~CI | 0.615 | 0.033 | 0.944 |
|  |  | ~IC*~PE*~PA*~PCV | 0.622 | 0.039 | 0.939 |
|  |  | ~IC*~VA*~PA*~CI | 0.606 | 0.055 | 0.938 |
|  |  | ~IC*~VA*~PE*~PCV*~CI | 0.577 | 0.026 | 0.952 |
|  |  | Solution | 0.736 | — | 0.907 |
|  | Parsimonious | ~IC*~VA*~CI + ~PE*~PA*~CI + ~IC*~PE*~PA | 0.776 | — | 0.884 |

Note. * = logical AND; ~ = absence. The intermediate solution was identical to the complex solution under the stated directional expectations. Configuration-level PRI values are reported in the main manuscript (Table 12).

**Table A14. Sensitivity analyses.**

| **Specification** | **Outcome** | **Intermediate solution** | **Coverage** | **Consistency** |
| --- | --- | --- | --- | --- |
| 0.501; PRI ≥ 0.70 | High | IC*VA*PE*PA*PCV + IC*VA*PE*PA*CI + IC*VA*PE*PCV*CI + IC*VA*PA*PCV*CI + IC*PE*PA*PCV*CI + VA*PE*PA*PCV*CI | 0.706 | 0.907 |
| 0.501; PRI ≥ 0.70 | Low | ~VA*~PE*~PA*~PCV*~CI + ~IC*~PE*~PA*~PCV*~CI + ~IC*~VA*~PA*~PCV*~CI + ~IC*~VA*~PE*~PCV*~CI + ~IC*~VA*~PE*~PA*~CI + ~IC*~VA*~PE*~PA*~PCV | 0.705 | 0.913 |
| 0.500 (unadjusted); PRI ≥ 0.65 | High | IC*VA*PE*PA + IC*VA*PE*PCV*CI + IC*VA*PA*PCV*CI + IC*PE*PA*PCV*CI + VA*PE*PA*PCV*CI | 0.712 | 0.903 |
| 0.500 (unadjusted); PRI ≥ 0.65 | Low | ~VA*~PE*~PA*~PCV*~CI + ~IC*~PE*~PA*~PCV*~CI + ~IC*~VA*~PE*~PCV*~CI + ~IC*~VA*~PE*~PA*~CI + ~IC*~VA*~PE*~PA*~PCV | 0.682 | 0.917 |

Note. With unadjusted 0.500 memberships, fsQCA reported tied prime implicants for both outcomes, so the parsimonious solutions are not unique; the main analysis therefore uses the 0.501 adjustment. No condition changed direction across specifications.

## **A.12 Notes on interpretation**

The stimulus should be understood as a controlled interpretive contact situation designed to give all respondents a consistent viewing experience. Because it was a five-page static storyboard, it did not reproduce the experience of a digital platform, immersive exhibition, interactive website, short video, AR guide, or gamified virtual heritage experience; this limitation is also acknowledged in the main manuscript.

## **A.13 Data and file linkage**

**Table A15. Data and file linkage.**

| **File** | **Content** |
| --- | --- |
| S1 Data | Anonymised item-level survey dataset for the 350 valid responses used in the CB-SEM and fsQCA analyses; submission timestamps, completion-time strings, source fields, IP addresses, and the original respondent sequence were removed or replaced by anonymous case identifiers. |
| S1 Appendix | This document. |
| S2 Code | Corpus cleaning, anonymisation, segmentation, TF-IDF, and auxiliary topic-model workflow. |
| Zenodo (https://doi.org/10.5281/zenodo.22919567) | The 3,433 initially collected records, the 2,332 excluded records, the 1,101 retained Chinese-language analytic records, the English-language companion corpus, retrieval strings, corpus-composition information, and metadata. |
